# Recognizing Acute Eosinophilic Pneumonia: A Rare and Often Overlooked Diagnosis

**DOI:** 10.51894/001c.165981

**Published:** 2026-07-31

**Authors:** Anne O’Connor, Michael Sukiennik, Radhika Edpuganti, Brendan Mamon, Natalia Baj-Osiewicz

**Affiliations:** 1 Henry Ford - Genesys

## 109

### Introduction

Acute eosinophilic pneumonia (AEP) is a rare and potentially life-threatening cause of acute respiratory failure, characterized by febrile illness, diffuse pulmonary infiltrates, and pulmonary eosinophilia.

### Case Description

We report a case of a previously healthy 20-year-old male who presented with a 3-day history of worsening shortness of breath, fevers, chills, dizziness, and a productive cough. Imaging findings showed diffuse bilateral pulmonary infiltrates. Peripheral eosinophilia was noted on manual differential several days into admission, suggestive of AEP. The patient received high-dose corticosteroids, along with antibiotic coverage for community-acquired pneumonia, with improvement in symptoms. Notably, the patient had reported recent initiation of cigarette smoking roughly one month prior to symptom onset, including a change in cigarette brands during this period.

### Discussion

This case highlights the diagnostic challenge of distinguishing AEP from other infectious etiologies of acute respiratory failure. Given the known association between smoking and AEP, this exposure may have played a contributory role. Early recognition and prompt corticosteroid therapy are critical to prevent morbidity.

